# Indigenous utilization of termite mounds and their sustainability in a rice growing village of the central plain of Laos

**DOI:** 10.1186/1746-4269-7-24

**Published:** 2011-08-18

**Authors:** Shuichi Miyagawa, Yusaku Koyama, Mika Kokubo, Yuichi Matsushita, Yoshinao Adachi, Sengdeaune Sivilay, Nobumitsu Kawakubo, Shinya Oba

**Affiliations:** 1 Faculty of Applied Biological Science, Gifu University, 1-1 Yanagido, Gifu, Japan; 2 Faculty of Agriculture, Shinshu University, Minamiminowa, Nagano, Japan; 3 The United Graduate School of Agricultural Science, Gifu University, 1-1 Yanagido, Gifu, Japan; 4 National Agriculture and Forestry Research Institute, Vientiane, Lao PDR

**Keywords:** Fertilizer, Laos, Mound volume, Paddy field, Termite, Termite mound

## Abstract

**Background:**

The objective of this study was to investigate the indigenous utilization of termite mounds and termites in a rain-fed rice growing village in the central plain of Laos, where rice production is low and varies year-to-year, and to assess the possibility of sustainable termite mound utilization in the future. This research was carried out from 2007 to 2009.

**Methods:**

The termites were collected from their mounds and surrounding areas and identified. Twenty villagers were interviewed on their use of termites and their mounds in the village. Sixty-three mounds were measured to determine their dimensions in early March, early July and middle to late November, 2009.

**Results:**

Eleven species of Termitidae were recorded during the survey period. It was found that the villagers use termite mounds as fertilizer for growing rice, vegetable beds and charcoal kilns. The villagers collected termites for food and as feed for breeding fish. Over the survey period, 81% of the mounds surveyed increased in volume; however, the volume was estimated to decrease by 0.114 m^3 ^mound^-1 ^year^-1 ^on average due to several mounds being completely cut out.

**Conclusion:**

It was concluded that current mound utilization by villagers is not sustainable. To ensure sustainable termite utilization in the future, studies should be conducted to enhance factors that promote mound restoration by termites. Furthermore, it will be necessary to improve mound conservation methods used by the villagers after changes in the soil mass of mounds in paddy fields and forests has been measured accurately. The socio-economic factors that affect mound utilization should also be studied.

## Background

Wet-season lowland rice production is an important rice production system in the plain areas along the Mekong River in the central region of Laos. The average harvested area and production of wet-season lowland rice from 1998 to 2007 were 296,790 ha and 1,012,801 t, comprising 79.1% and 77.6%, respectively, of the total harvested area and production in the central region. Dry-season lowland rice and wet-season upland rice constitute the remainder of the area and production [1]. Wet-season rice is grown usually in rain-fed paddy fields because of the limited area of irrigation close to the Mekong River and its tributaries. Therefore the yield is lower and more variable among paddy plots and year-to-year than in irrigated paddy fields [2,3]. The lower fertility of paddy field soils than that in the northern region is another unfavorable factor for rice production [4]. Accordingly, rice is seldom sold except in particularly productive years. Chemical fertilizer, improved cultivars and tillage machines for rice cultivation are procured depending on cash income from the gathering and sale of natural resources and off-farm jobs in urban areas [3,5].

In the plain areas of Laos as well as in Northeast Thailand, which is adjacent to the plain areas of Laos beyond the Mekong River, many trees and termite mounds were reported [6-8]. The results of the rice yield survey in a village of the central plain of Laos suggested that higher yields near trees than in open areas were due to the presence and development of termite mounds around trees in the paddy fields [9]. We have observed that villagers used the termites for food and as feed for breeding fish, and have used their mounds for vegetable seedling beds, fertilizer in paddy fields, and charcoal kilns among other uses. Many reports have mentioned the possibility of using termite mounds for fertilizer and have described examples of its use in crop fields in Africa [10-14]. Soil from the mounds is also used for construction materials in northwestern Namibia [15]. In Northeast Thailand, higher crop productivity at the site of leveled termite mounds was analyzed [16], and the use of mound soil as fertilizer in intensive vegetable production was estimated to be high [17]. However, other than our observation in Laos, little information is available on the indigenous utilization of termite mounds for agriculture and other forms of livelihood in rural areas. If termite mound soil is available in sufficient quantity, it can be used as fertilizer for rice growing in a village to enhance rice yield without purchasing chemical fertilizer. However, in the rice growing villages of Laos, the indigenous utilization of termites and termite mounds has not been studied, nor has the possible future sustainability of their use been evaluated. The objective of this study is to evaluate the possibility of sustainable use by measuring the change in volume of selected mounds and by investigating how villagers actually use termite mounds.

## Methods

This study was carried out in Dong Khuai village (18°01' N, 102°48' E) in the Vientiane capital, Lao P.D.R. from 2007 to 2009. Dong Khuai is a typical rain-fed rice growing village on the Vientiane Plain. Ninety-three percent of the 255 households were engaged in rain-fed rice production. The village area is 2,528 ha, including 820 ha of paddy fields. A paddy field area of 134.8 ha (6,883 plots), which is located on a gently sloping plain around the village settlement, was selected for the present study.

We collected the soldiers of termites by hand from the surface, underground and the area surrounding the mounds including standing trees in the study area in August and November in 2007, from October to December in 2008 and March in 2009. Their species were identified by a specialist referring to Ahmad [18].

Twenty villagers engaged in farming with many years of experience who were willing to cooperate in the study were selected from households and interviewed on the usage of termites and their mounds in the village. Information was gathered on how termites and their mounds and mound soil were used, the effects on crop performance, and the estimated volume of mounds used annually. Interviews were carried out in the local language by native Lao speaking members of the research team.

Sixty-three mounds were selected from the 383 mounds in the study area to determine their dimensions. This excludes mounds that were damaged while collecting termites for species identification. Mounds were selected to include various sizes and to account for even distribution in the study area, having obtained permission from the villagers. Mounds with dense tree bush were not selected as the bush obstructed measurement. In this report, we show the average dimensions of sampled mounds in the study area without taking into account the termite species. The height and base circumference at ground level of each mound were measured and then photographs were taken from two or more angles for each measurement in early March, early July and mid to late November in 2009 during the periods agreed with the villagers. The mound volume was calculated from the photographs using 3D photogrammetry software (Kraves K, Kurabo Industrial Ltd.).

## Results and discussion

### Termite species

We identified 11 termite species belonging to the family Termitidae (Table 1). Seven species (Nos. 1, 2, 4, 7, 8, 10 and 11) were found in the mound soil. Other species were found on the surface of the mounds and/or tree trunks. Some mounds contained multiple species. We aimed to avoid the destruction of mounds for species identification, and thus were unable to determine the proportion of the 63 mounds measured that were made by each species.

**Table 1:** Species of Termitidae found in the surveyed area

**No**.	Subfamily	Genus	Species
1	Macrotermitinae	Macrotermes	*Macrotermes gilvus*
2		Odontotermes	*Odontotermes feae*
3			*Odontotermes *sp *1*.
4			*Odontotermes *sp *2*. similar to *jabanicus*
5		Microtermes	*Microtermes obesi*
6			*Microtermes *sp.
7	Amitermitinae	Microcerotermes	*Microcerotermes crassus*
8		Globitermes	*Globitermes sulphureus*
9	Nasutitermitinae	Hospitalitermes	*Hospitalitermes ataramensis*
10	Termitinae	Pericapritermes	*Pericapritermes latignathus*
11		Termes	*Termes propinquus*

The mound distribution and mound formation activity of individual species is beyond the scope of this report, and should be studied in fields where there is no economic activity by villagers.

### Indigenous utilization

**Figure 1:**
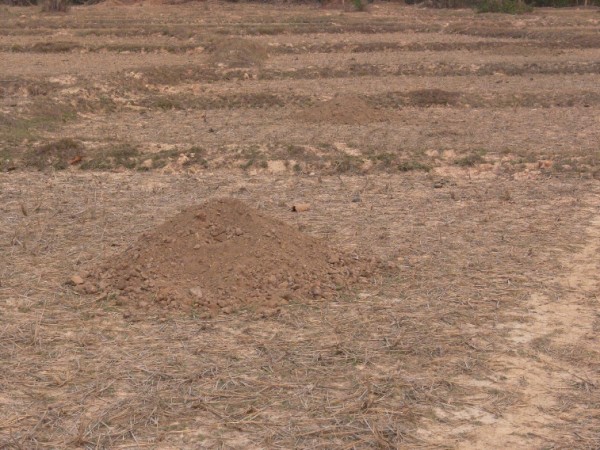
**Termite mound soil placed in paddy field before plowing**. Width of soil mound was ca. 80 cm. Termite species was unknown.

**Figure 2:**
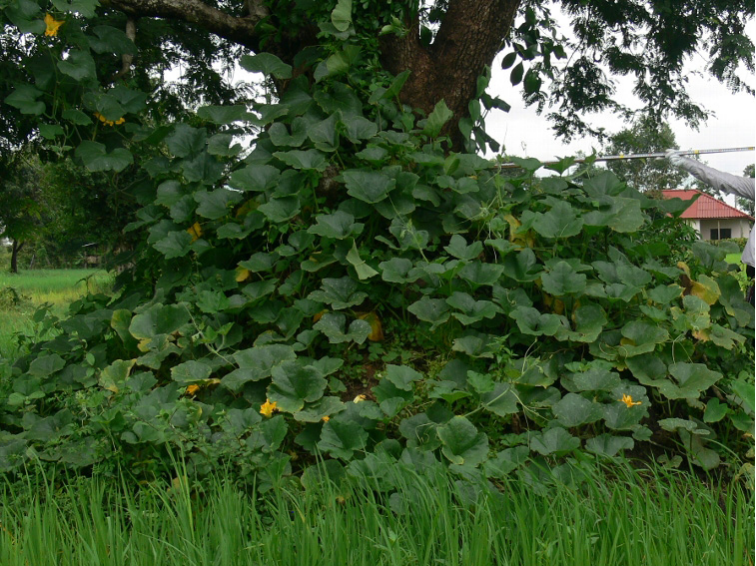
**Termite mound for vegetable (pumpkin) growing**. Base diameter and height of mound were ca. 3 m and ca 1.5 m, respectively. Termite species was unknown.

**Figure 3:**
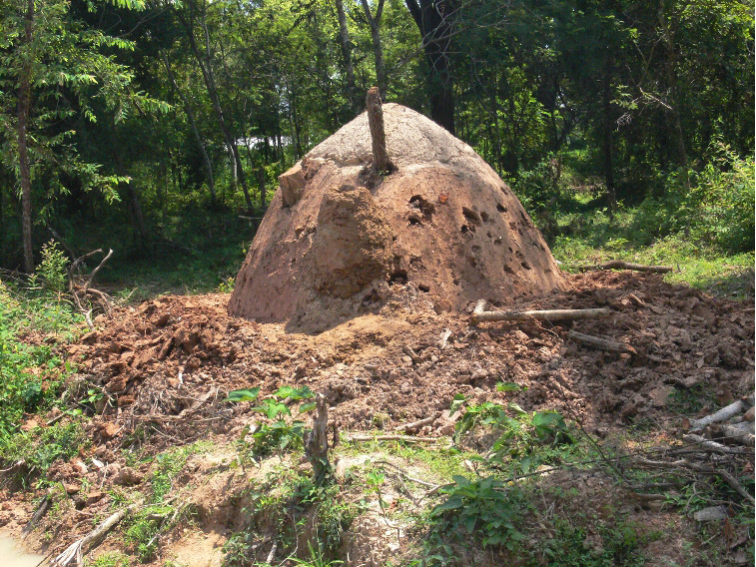
**Termite mound for charcoal kiln**. Base diameter and height of kiln were 2.4 m and 1.8 m, respectively. Termite species was unknown.

**Figure 4:**
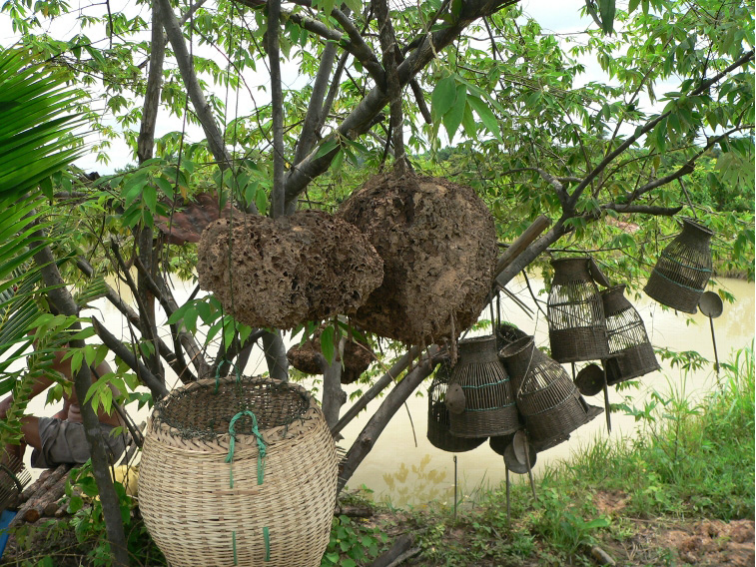
**Termites in mound soil prepared for fish feeding**. Termite species was unknown.

According to the interviews, none of the villagers sold or gave away mound soil from their own land. Therefore it can be said that mound soil is used primarily for self-sufficiency in this village. All respondents had used mound soil as fertilizer for rice, and 95% of them used it every year. They spread the soil in paddy fields or upland rice fields in May and June before rice planting. Mound soil was not used as fertilizer for any crop other than rice. The villagers dig the mounds with hoes, crush lumps of mound soil, and then convey it to the field where it is spread (Figure 1). The soil is mixed with paddy field surface soil during plowing. Some respondents used 2 to 4 mounds in a year, but we were unable to determine accurately the amount used by each respondent. Ten percent of the respondents grew vegetables on mounds because the soil is thought to be fertile and have good drainage for vegetable growth (Figure 2). Their assertion was that rice growth is better near mounds in paddy fields than in areas without mounds. Sixty-five percent of the respondents used mounds for charcoal kilns (Figure 3). Eighty percent used termites as fish feed mostly for catfish (*Claris *sp.) and snakehead fish (*Channa striata*), which are bred in ponds near to the work huts in paddy fields. The amount of mound soil used when extracting termites for feeding fish varied widely among the respondents, ranging between 2 kg and 1000 kg in a year. The soil is removed from the mounds and the soil lumps are crushed to remove the termites, which are then fed to the fish (Figure 4). Some respondents also used the termites as fishing bait and chicken feed. No other forms of mound soil utilization were found.

**Figure 5:**
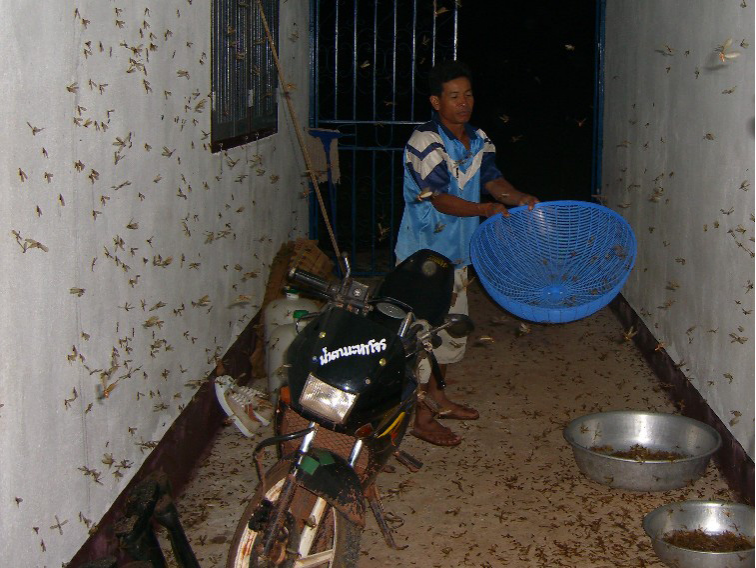
**Catching emerged termites for eating**. Termite species was unknown.

It was usually observed in the early rainy season that villagers catch emerged termites (alate) and eat them after broiling (Figure 5). Wherever the alate first land, they immediately lose their wings. Villagers use water basins to catch the termites and prevent them from escaping. They also collect termite mushrooms (*Termitomyces *sp./spp.) for eating and for sale on markets. The mushrooms are the most prized wild mushrooms in the Vientiane Plain due to their flavor [19]. Various kinds of natural resources are used by the villagers, most of which are sold on markets to earn cash [20].

### Changes in termite mound volume

The average height, base circumference at ground level and mound volume were 90 to 95 cm, 479 to 566 cm and 1.1 to 1.2 m^3 ^respectively, but these dimensions varied considerably among mounds (Table 2). Some mounds were completely cut out after March, and some were not measured due to the hazard presented by bees. These differences in dimension may have been due to the years of habitation, population size, termite species, the degree of weathering and the extent of use by villagers. Further study is required.

**Table 2:** Dimensions of the termite mounds surveyed

Survey period		Height (cm)	Base circumference at ground level (cm)	**Volume (m**^**3**^)
March	N	62	63	58
	Mean	93	479	1.14
	Maximum	284	1592	11.22
	Minimum	26	27	0.00
	SD	53	364	1.72
				
July	N	49	49	47
	Mean	91	536	1.16
	Maximum	190	1663	11.27
	Minimum	26	110	0.01
	SD	43	293	1.86
				
November	N	52	51	47
	Mean	95	566	1.17
	Maximum	205	1445	11.27
	Minimum	36	145	0.01
	SD	47	316	1.86

* Excluding mounds with no volume after being completely cut out.

**Figure 6:**
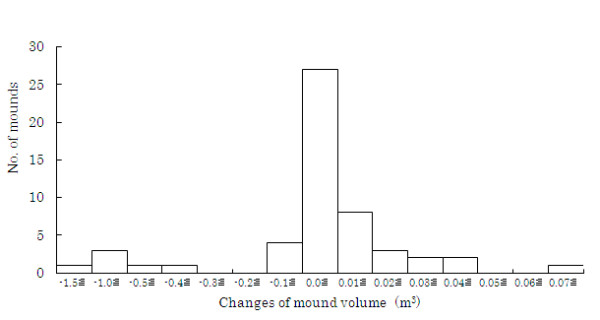
**Frequency of changes in mound volume from March to November, 2009**.

The volume of 53 mounds, including mounds that had been cut, was measured individually 3 times from March to November (Table 3). The average volume increase was -0.077 m^3 ^from March to July and 0.002 m^3 ^from July to November. The negative growth in the former period may have been caused by the cutting out of mounds for use as fertilizer and making charcoal kilns. The positive growth in the latter period was due to the restoration of the mounds by the termites. Observation has shown that termites restore their mounds quickly after they are destroyed by villagers. However, the average mound volume in the surveyed area decreased by -0.076 m^3 ^from March to November. During the survey period, 81% of the mounds increased in volume and the frequency of mounds that increased in volume by 0 to 0.01 m^3 ^was the highest among all mounds, possibly as a result of restoration by termites (Figure 6). Mounds that decreased in the volume range of -1.5 to -0.4 m^3 ^corresponded to those completely cut out by villagers. The small decrease in the range -0.4 to 0 m^3 ^probably resulted from mounds being partly cut out by villagers and/or weathering. Such factors contributed to a substantial reduction in mound volume compared with the increase resulting from termite restoration activity. We were able to determine the gross reduction in mound size only due to complete destruction and not due to use by the villagers or other factors. Thus, the gross size increase due to termite activity is also unknown. These factors should be studied in detail over a long period in the future after gaining the villagers' permission to conduct the survey. Control mounds preserved from destruction would be necessary to enable the accurate measurement of mound growth. Further, restoration after artificial destruction should also be measured. Such studies will be possible in natural fields outside of the village area.

**Table 3:** Changes in the mound volume (m^3^) during the survey period

	March-July	July-November	March-November
N	53	53	53
Mean	-0.077	0.002	-0.076
Maximum	0.092	0.190	0.079
Minimum	-1.336	-0.090	-1.336
SD	0.266	0.034	0.266

### Evaluation of sustainable utilization of termite mounds

The net growth of termite mounds was estimated from the results in Table 3 as -0.009 m^3 ^per month. When the growth rate is equivalent to the average over the whole year, the net growth rate in volume may be -0.114 m^3 ^mound^-1 ^year^-1^. Since termite mound density is estimated to be 1.41 ha^-1 ^in the paddy field area of 134.8 ha around the village settlement, the total volume increase will reach -120.0 m^3 ^per year in the whole paddy field area of 749.4 ha of this village. The total volume may correspond to 79.7 mounds of average size. Therefore the termite mounds in paddy fields are declining and may disappear in the future. However, the villagers' use of mounds in areas far from the village settlement may not differ from that in the studied area. Moreover, the presence of more termite mounds in forests and grassland areas has been observed, although their volume was not measured in this study, and the villagers' use of these mounds will be less frequent than those in the paddy field area. Therefore, a reduction in mound volume of less than 120.0 m^3 ^may be expected in this village. However, the results suggest that the sustainable utilization of termite mounds is difficult at this time and will become impossible in the future, even if the intensity of mound utilization does not increase from its current level. Many farmers have recently been using tillage machines for cultivation. These farmers tend to destroy the termite mounds, complaining that the mounds make it difficult to maneuver the tillage machines in the paddy fields. Such issues should be studied further to limit destruction and enhance the factors favorable to mound restoration by termites. For example, varying the species used, locations, extent of destruction and improving conservation methods could enable sustainable utilization in the agro-ecosystem, thereby promoting a symbiosis between the villagers and termites. We often observed preserved mounds in yards of residential areas.

Termite mushrooms are a highly sought after food and have become an important cash crop for the villagers [19]. Such demand may help to control the rapid destruction of mounds. The socio-economic situation and the villagers' intentions that affect the use and preservation of termites in the village should be also studied.

## Conclusion

This study showed that termites and termite mounds are used in various ways in a rice growing village in the central plain in Laos. The mounds are used not only for fertilizer as previously reported in other countries, but also as beds for growing vegetables and for making charcoal kilns, depending on the shape of the mound. However, further investigation is required to clarify differences in the utilization of termites and termite mounds among the various termite species. Our observations suggest that current mound use by villagers is unsustainable. Once all the termite mounds have been completely used and destroyed in this area, the traditional knowledge of termites and termite mound utilization, including termite mushrooms, will be lost. In order to propose a plan for the conservation and sustainable utilization of termites, it is necessary to accurately measure changes in mound soil mass in paddy fields and forests, and further develop non-destructive methods of collecting termites to identify species. Further, the socio-economic factors affecting utilization-related activities should be studied in the near future.

## Competing interests

The authors declare that they have no competing interests.

## Authors' contributions

SM drafted the theoretical framework for the discussion. YK and OS measured termite mound dimensions. MK and NK collected and identified termites. YM investigated the geographical distribution of mounds. YA and SS carried out interview work and collected information on indigenous knowledge. All authors discussed the data, read and approved the final manuscript.
